# Structural Properties and Damage Detection Capability of Carbon Nanotube Modified Mortars after Freeze-Thaw

**DOI:** 10.3390/ma12111747

**Published:** 2019-05-29

**Authors:** Panagiota Alafogianni, Ilias Tragazikis, Anastasios Balaskas, Nektaria-Marianthi Barkoula

**Affiliations:** Department of Materials Science and Engineering, University of Ioannina, 45110 Ioannina, Greece; pan.alafogianni@gmail.com (P.A.); iliastra@yahoo.gr (I.T.); tbalask@gmail.com (A.B.)

**Keywords:** CNTs, dispersive agents, freeze-thaw, fracture, damage monitoring

## Abstract

Here we explore the structural properties and damage sensing of cementitious mortars after a freeze-thaw process (F-T) as a function of nano-modification. For this purpose, carbon nanotubes were added at 0.2–0.8 wt.% cement using two different dispersive agents. F-T resulted in reduced fracture energy in nano-modified specimens prepared using superplasticizer as a dispersant while the opposite held true for the surfactant-containing ones. All nano-modified mortars possessed significantly higher fracture energy compared to the plain specimens after F-T (up to 73% improvement). The acoustic emission activity was lower after F-T, while acoustic emission indicators revealed a more tensile mode of fracture in both plain and nano-modified mortars.

## 1. Introduction

Over the last two decades, carbon nanotube (CNT) modified cementitious composites [1,2,3,4,5,6] have been investigated with focus on two main aspects: a) the improvement of their performance and b) the production of multifunctional, smart and self-sensing materials [7,8,9]. The ability of CNTs to bridge micro-cracks and delay their propagation [10,11,12] has been proven beneficial for the fracture behavior of cementitious materials [13,14]. It has been documented that the fracture strength, toughness as well as flexural toughness index is considerably increased after the addition of low amounts of single- and multi-walled CNTs in cementitious pastes and mortars [3,13,15,16,17]. Furthermore, mechanical and fracture studies combined with acoustic emission (AE) measurements also demonstrate the enhanced damage sensing capability of cementitious materials modified with carbon nano-inclusions [18,19]. 

An environmental parameter of huge significance for the fracture behavior and integrity of cementitious materials is the freeze-thaw (F-T) cycling encountered in cold environments. F-T can damage cementitious materials by creating internal cracking due to cold temperature cycling. When water freezes, it exhibits a 9% volume increase that may cause deterioration either to the hardened paste, the aggregates, or both [20]. Since water is contained within the pores of the cementitious matrix destructive tensile stresses varying between 1N/mm^2^ and 4N/mm^2^ may be generated, depending on the exposure temperature, the connectivity of the pores and the degree of saturation [21,22,23]. This may result in internal micro-cracks at an early exposure stage while repeated F-T cycles may cause permanent damage to the material. The extent of deterioration depends on the conditions of exposure of the material and its transport properties, i.e. its water absorption profile. Thus, damage due to F-T may occur with low-temperature cycling combined with a high degree of saturation (>86%) [24,25]. 

Several studies suggest a beneficial role of carbon-based nano-inclusions on the microstructure [3,4,5,6,7,17,26,27] and in turn water sorption and water permeability [28,29,30] of cementitious materials. This has been mainly attributed to the refinement of pores and reduction in pore connectivity and bridging. Since pore structure and water permeability are of enormous significance for the F-T response of cementitious materials the addition of CNTs is also expected to greatly affect their performance after F-T cycling [31]. Indeed, recent studies demonstrate a considerable increase of the mechanical properties and dimensional stability of cementitious composites after the addition of small amounts of CNTs [32,33,34]. It has been suggested that the optimized performance of CNT-modified cementitious materials is linked with the aforementioned bridging effect of CNTs as well as the decreased fraction of large-capillary pores caused by the incorporation of the nano-phase inclusions [33]. Elsewhere however a more rapid deterioration of the relative dynamic modulus of CNT-modified composites after F-T cycling is presented in comparison to un-modified specimens [35]. 

It is well accepted that the dispersion of CNTs is of huge significance for the performance of cementitious materials. Insufficient dispersion has been related to the agglomeration of CNTs and in turn, inadequate stress transfer, crack initiation due to stress concentration around agglomerates and limited sensing potential [36]. CNTs are commonly dispersed by an agent (surfactant or plasticizer) in the water of the cementitious mixture, a procedure which is normally supported by the application of sonication [2,3]. The selection of type/concentration of dispersive agents is however not straightforward due to limitations in the dispersion ability of plasticizers and substantial increase of the air content values in the case of surfactants [37]. Quite recently we have demonstrated that the nature of dispersive agents determines the microstructure, absorption and barrier properties of CNT-modified cementitious mortars [37]. 

Based on the above, the present study contributes to current knowledge by exploring the effect of F-T on the structural properties and damage detection capability of nano-modified cementitious mortars. The dynamic modulus of elasticity, compressive strength, and fracture energy were examined before and after F-T cycling while damage sensing was monitored via AE measurements. Two different agents were used to support CNT dispersion in the cementitious matrix, one belonging to the surfactant and the other to the superplasticizer family. CNT content varied between 0 and 0.8 wt.%, in order to cover the range below and above the multi-functionality threshold of approximately 0.5–0.6 wt.% [9,38].

## 2. Materials and Methods

### 2.1. Materials

CNTs (“ONEX MW1000C1”) with a 20 to 45 nm diameter and a length over 10 μm were purchased from Glonatech SA, Athens, Greece. Two dispersive agents were used in the current study, an anionic surfactant (sodium-dodecyl-benzene sulfonate, Sigma-Aldrich, Taufkirchen, Germany) particularly popular for CNT separation and an anionic, polycarboxylate based superplasticizer (Viscocrete Ultra 300, Sika Hellas SA., Krioneri, Greece) specific to suspensions targeted for nano-modified cementitious materials [37]. Portland cement type I and natural sand were used for the preparation of the cementitious mortars. When necessary, tributyl-phosphate (TBP) (Sigma-Aldrich, Taufkirchen, Germany) and Viscocrete ultra 600 (Sika Hellas SA., Krioneri, Greece) were applied to adjust the air content and workability of the fresh mortars, respectively. 

### 2.2. Preparation of Nano-Modified Mortars

**Figure 1:**
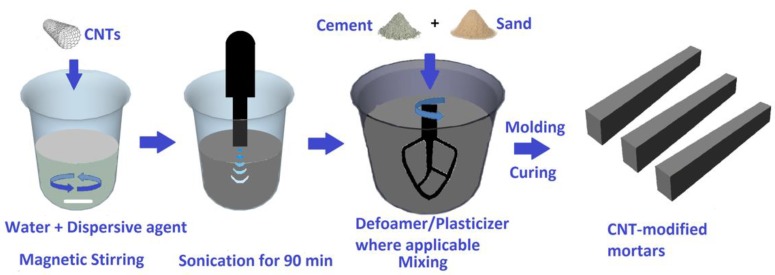
Schematic illustration of the specimen’s preparation procedure.

BS EN 196-1 was followed for the preparation of the mortar specimens. Specimen’s preparation procedure is schematically illustrated in Figure 1, while details on the preparation procedure can be found elsewhere [37]. In brief, the water to cement (w/c) ratio was set at 0.5 while the sand to cement (s/c) ratio was 3. A constant amount of cement (450 g), water (225 g) and sand (1350 g) were used for the preparation of all mortars. First, CNTs (0.2–0.8 wt.% of cement) were dispersed in the water of the mixture following a procedure developed by members of our research group [36,39]. For optimum dispersion and limited alteration of the physical properties of the prepared mortars, the surfactant’s concentration was kept as low as 50% of CNTs, while the respective amount of superplasticizer was as high as 150% [36,38,39]. CNT-based suspensions were then mixed together with cement and sand for the preparation of the nano-modified mortars. In the case of surfactant-assisted mixtures, a constant amount of TBP (0.7 g) was also added together with the CNT-based suspensions. Workability was fixed by adding an appropriate amount of Viscocrete Ultra 600 (up to 2 g) at the end of the mixing procedure. Mixtures were molded for 24h and then demolded and stored in a humid environment for 27 days.

### 2.3. Freeze-Thaw Cycling

F-T conditions were simulated using an environmental chamber (Vötsch VC 4018, Vötsch Industrietechnik GmbH, Balingen, Germany) according to ASTM C666 [40]. Six saturated specimens of each composition were placed in the chamber and subjected to 300 F-T cycles. Each cycle included 4 h of freezing at −18 °C (dry conditions) and 4 h of thawing at 4 °C (relative humidity approximately 98%). An equal number of un-modified and CNT-modified mortars were stored in a laboratory environment for reference (un-exposed specimens, designated as “before F-T” specimens). 

### 2.4. Testing Procedure

Ultrasound experiments were conducted on un-modified and CNT-modified mortars employing R15a AE sensors (Physical Acoustics Corp, Princeton, NJ, USA), fixed on the two opposite sides of the mortar beams [19]. The dynamic elastic modulus, E, was calculated according to Equation (1):

(1) $$C_{p}=\sqrt{\frac{E_{d}\left(1-\nu \right)}{\rho \left(1+\nu \right)\left(1-2\nu \right)}}$$

where *Ed* is the dynamic elastic modulus in GPa, *C_p_* is the longitudinal wave velocity in m/s, *ρ* is the density of mortar beams in Kg/m^3^, and *ν* the Poisson’s ratio. 

Compression tests were conducted according to BS EN 196–1:2005 standard [41] on a MATEST mortar testing machine (Matest, Arcore, Italy) with a loadcell of 250 kN. Cubic specimens (40 × 40 × 40 mm^3^) were tested at a rate of 1.5 MPa/s.

**Figure 2:**
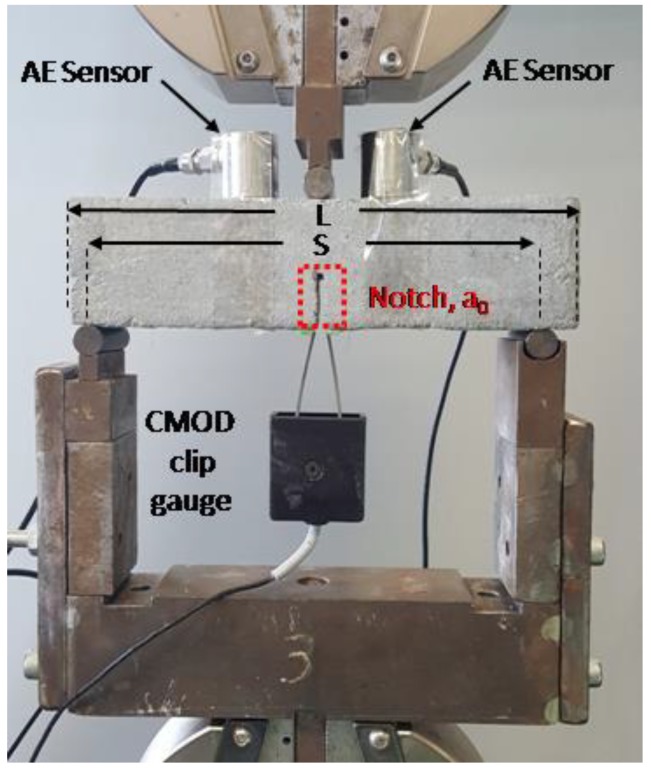
Configuration of the fracture energy test set-up.

The fracture energy of the un-modified and CNT-modified mortars was assessed using notched beams as illustrated in Figure 2, according to Reference [19]. The notch on each prismatic specimen (40 × 40 × 160 mm^3^) was cut to half of the prism’s height (i.e., 20 mm) [19]. Three-point bending experiments were conducted on an Instron 5967 (Instron, Norwood, MA, USA) 30 kN testing machine at a speed of 0.02 mm/s. An Instron clip on gauge was used to monitor the crack mouth opening displacement (CMOD) of the notch. Details on the test configuration and experimental set-up are provided elsewhere [19,42]. The fracture energy (G_f_) in N/m of all specimens was calculated using Equation (2) [19,42]:

(2) $$G_{F}=\frac{W_{o}+mg\delta_{o}}{A_{lig}}$$

where *W_o_* is the area under the load-CMOD curve, *m* is the weight of the device participating in the experiment (kg), *g* is the acceleration due to gravity (9.81 m/s^2^), *δ_o_* the CMOD of the beam at fracture (m), and *A_lig_* is the area of the ligament (m^2^) [19,42]. 

Damage monitoring was performed using two R15a AE sensors, with max sensitivity at 150 kHz, attached on the specimen at a distance of 40mm, as illustrated in Figure 2. Details on the experimental set-up can be found in our previous publication [19]. 

Statistical calculations (mean values and standard deviation) were performed on the results of six specimens.

## 3. Results

**Figure 3:**
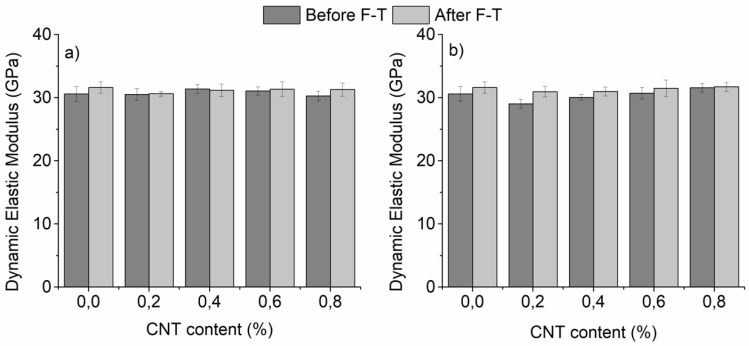
Dynamic elastic modulus of nano-mortars prepared with (**a**) superplasticizer and (**b**) surfactant as a dispersive agent, as a function of F-T exposure (plain specimens have been added for reference).

The dynamic elastic modulus of un-modified and CNT-modified mortars is presented in Figure 3 as a function of F-T exposure. The obtained values show little variation with CNT content, and F-T cycling when superplasticizer is the dispersant (Figure 3a). For nano-modified mortars with surfactant, a slight increase was observed in the dynamic modulus after F-T cycling (Figure 3b). 

The same holds for the un-modified specimens. This behavior could be linked with the continuation of the hydration process during F-T in the case of un-modified and CNT-modified specimens with surfactant. Ιn specimens where superplasticizer was used as dispersant it was speculated that hydration was completed prior to F-T exposure. Improved hydration, even after 7 days of curing, has been documented in the past in cementitious mortars with polycarboxylate-based superplasticizers [43,44], while the opposite has been observed in surfactant-containing specimens [45,46]. 

**Figure 4:**
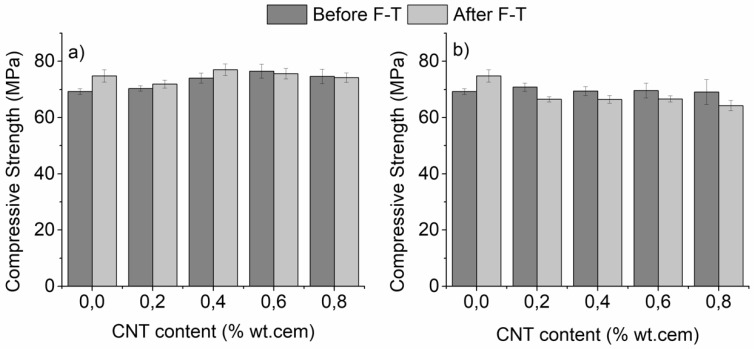
Compressive strength of nano-mortars prepared with (**a**) superplasticizer and (**b**) surfactant as dispersive agent, as a function of F-T exposure (plain specimens have been added for reference).

The compressive strength of un-modified and CNT-modified mortars before and after F-T is presented in Figure 4a for specimens with superplasticizer and Figure 4b for those with a surfactant as a dispersive agent. Figure 4a shows an up to 10% enhancement of the compressive strength, which finds its maximum after the addition of 0.6% CNTs before F-T exposure. As observed, further addition of CNTs results in a reduction of the compressive strength. This phenomenon has been documented before and has been linked with nanotube agglomeration at higher loading fractions [18]. Interestingly, F-T cycling results in a further increase of the compressive strength of specimens without and with low amounts of CNTs (up to 0.4%) while the strength is not negatively affected at higher CNT contents after F-T when superplasticizer was applied as a dispersant (Figure 4a). Independent to the CNT content, surfactant-containing specimens (Figure 4b) did not show a significant enhancement of their strength before F-T. At the same time, the compressive strength slightly dropped in all nano-modified specimens after F-T (Figure 3b). It is well accepted that the compressive strength of the cementitious matrix is related to the w/c ratio, the level of compaction and the porosity of the matrix [47]. Since compressive loading does not support extensive crack mouth opening, micro-cracks that developed during the F-T stage were not expected to substantially propagate during compression and thus, the behavior presented in Figure 4 should be more linked to the effect of F-T on the microstructure of the matrix. It has been previously documented that small amounts of nano-modification lead to a reduction in the number of capillary pores in both surfactant and superplasticizer containing specimens [37]. The reduction was, however, higher in nano-modified mortars using superplasticizer as a dispersant [37]. Another factor that needs to be considered is the macro-porosity in surfactant-containing specimens due to the air-entrapment action of the surfactant. As aforementioned, TBP was used during the preparation of the surfactant-containing specimens in order to keep the air content values constant. However, it was speculated that air was locally entrapped in the cementitious matrix leading to as light deterioration of the compressive strength. Based on the above, it can be understood why the compressive strength of CNT-modified specimens prepared with superplasticizer was superior to those prepared using surfactant, prior and post F-T cycling.

**Figure 5:**
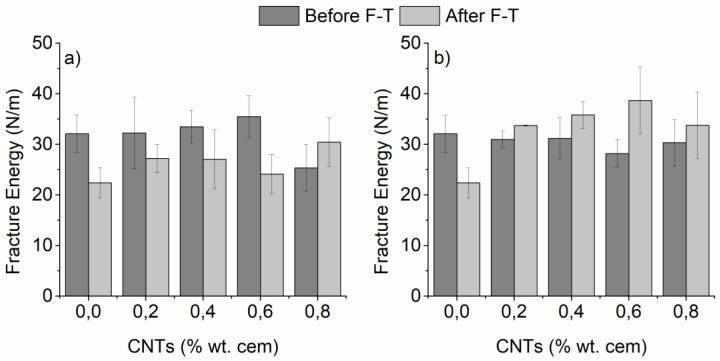
Fracture energy of nano-mortars prepared with (**a**) superplasticizer and (**b**) surfactant as dispersive agent, as a function of F-T exposure (plain specimens have been added for reference).

Since the opening of a crack is not supported during compression, it is imperative to evaluate the effect of F-T on the fracture behavior of the nano-modified mortars under bending conditions, which facilitate the crack propagation. This effect is illustrated in Figure 5a,b, based on the energy required to fracture notched specimens prepared using a superplasticizer and surfactant as dispersive agents, respectively. Nano-modified mortars prepared with the aid of superplasticizer presented a slight increase in the fracture energy before F-T cycling, with the exception of specimens with 0.8 wt.% CNTs which showed an approximately 20% drop in the fracture energy(Figure 5a). Surfactant-based mortars maintained their fracture energy for CNT contents up to 0.6 wt.% before F-T, while further addition resulted in a slight deterioration of the energy (Figure 5b). This could be due to the insufficient dispersion of CNTs at high CNT loadings. Agglomerates acted as stress concentration points, which facilitated the generation and propagation of cracks and in turn resulted in reduced fracture energy.

It is worth mentioning that after F-T, the response of nano-modified mortars presented opposite trends under bending loading compared to compression. Nano-modified specimens prepared with superplasticizer showed a considerable reduction (up to 32%) of the fracture energy after F-T exposure when the CNT content was as high as 0.6 wt.% while mortars modified with 0.8 wt.% CNTs displayed an approximately 20% increase in the fracture energy. On the contrary, surfactant-assisted nano-modified mortars demonstrated enhanced fracture behavior after F-T exposure for all CNT contents, while mortars with 0.6 wt.% CNTs displayed the highest improvement (approximately 37% increase of the fracture energy compared to the same specimens before F-T cycling). Another interesting observation was that all nano-modified mortars possessed a much higher fracture energy compared to plain specimens after F-T (up to 73% improvement). Thus it was obvious that nano-modification was beneficial for the durability of cementitious mortars exposed to F-T conditions. Furthermore, it can be concluded that the dispersive agent-related microstructure played a substantial role in the fracture behavior of nano-modified mortars. 

**Figure 6:**
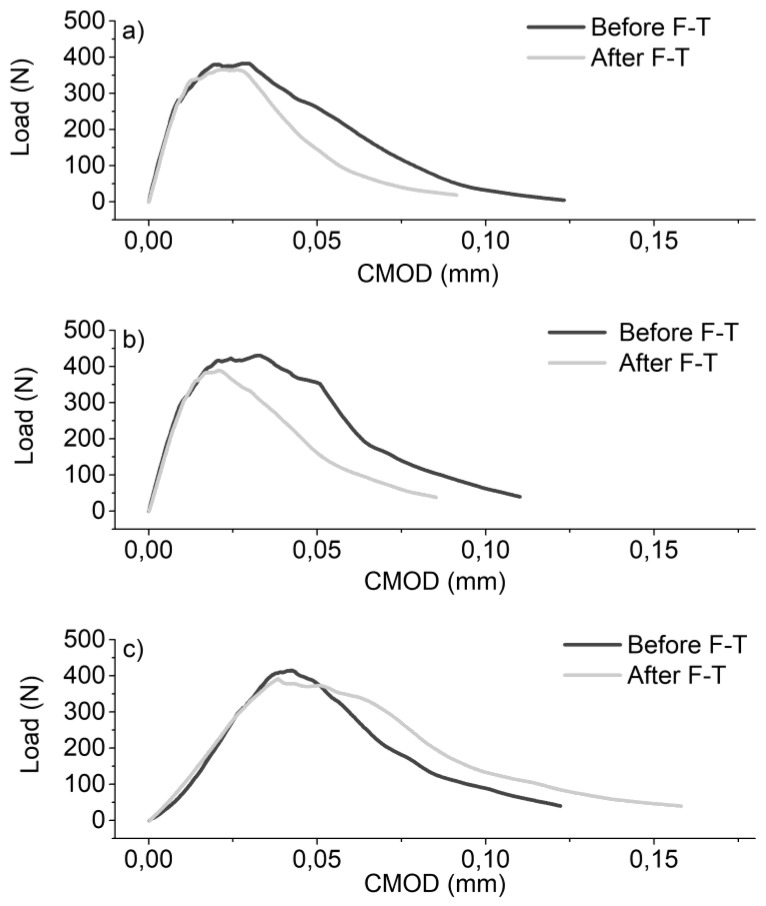
Load-crack mouth opening displacement (CMOD) graphs of unmodified (**a**) and 0.6% nano-modified mortars prepared with (**b**) superplasticizer and (**c**) surfactant as a dispersive agent, as a function of F-T exposure.

In order to elucidate on these findings, a direct comparison of the representative load-CMOD curves is provided in Figure 6, based on the example of specimens with 0.6 wt.% CNTs. As illustrated in Figure 6a–c, the elastic region of the load-CMOD curves was not altered due to F-T cycling in both un-modified and 0.6 wt.% CNT-modified mortars. On the contrary, plastic deformation was considerably affected by F-T cycling. Un-modified and modified specimens with 0.6 wt.% CNTs prepared using superplasticizer as a dispersant displayed lower fracture toughness after F-T, as illustrated by the reduction of the area under the load-CMOD curve compared to that before F-T. On the other hand, surfactant-containing specimens modified with 0.6 wt.% CNTs showed a slightly lower peak load but considerably higher plastic deformation, indicative of their higher toughness after F-T. The elastic response was determined by the stiffness of the prepared mortars, while their plastic deformation was linked with the initiation, propagation and bridging of cracks as well as pull-out of the reinforcement. 

Thus, in order to explain the behavior presented in Figure 5 and Figure 6 we need to consider the crack formation and propagation during F-T, and how this is influenced by CNT dispersion with the aid of dispersive agents. The main driving force for crack formation is F-T of water. As mentioned above, the pressure that builds up during F-T cycling depends greatly on the amount of absorbed water as well as the size and the connectivity of the pores. Overall the enhanced fracture response of nano-modified specimens after F-T compared to un-modified ones could be explained on the basis of pore refinement, and thus reduction of contained water, as well as bridging of cracks due to the dispersion of CNTs. Previous studies have demonstrated that the total amount of water after full immersion was lower in nano-modified specimens prepared with a surfactant as a dispersive agent compared to those prepared with superplasticizer [37]. As explained in Reference [37], both sodium-dodecyl-benzene sulfonate and Viscocrete contain hydrophilic groups (sulfonate groups of the former, hydroxyl- and carboxyl-groups of the later) that may interact with water. However, due to the higher amount of superplasticizer based specimens were more susceptible to water than surfactant ones. Furthermore, as aforementioned, it was documented that specimens prepared with superplasticizer presented smaller gel-sized pores compared to the medium-sized pores of surfactant specimens [37]. Taking these two facts into consideration, it can be speculated that compared to nano-modified specimens using surfactant, those prepared using superplasticizer hold more water which cannot be freely expanded since it is contained in smaller-sized pores, leading thus in higher build-up pressures. This argument is in line with the results of atomistic modeling and molecular dynamic simulations on water desorption/adsorption during F-T cycles, which revealed increased nano-pore failure in graphene reinforced cementitious materials due to water movement between gel and capillary pores [48]. Thus, the enhanced performance of surfactant-containing specimens after F-T could be associated with a lower amount of water and ease of water migration from medium-sized to capillary pores during F-T, which results in a lower amount of nano-pore failure. Additionally, as already discussed, although an antifoaming agent (TBP) has been used in surfactant-containing specimens, it was expected that air was locally entrained, improving the F-T resistance of these specimens. Overall it can be concluded that next to pore refinement due to nano-modification, one should consider also the type of dispersive agents used for the preparation of the nano-modified specimens, the air forming behavior and their affinity with water in order to access their F-T response.

**Figure 7:**
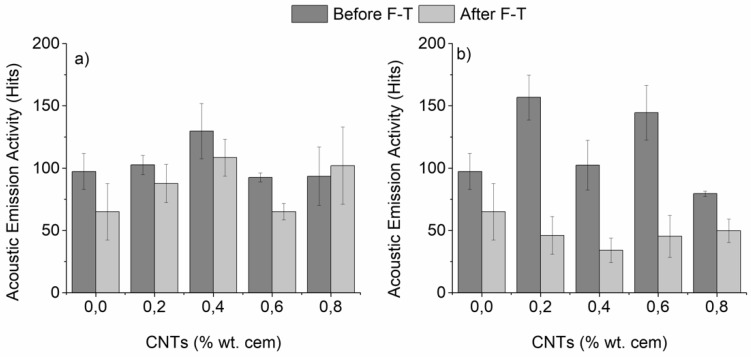
Acoustic emission activity of nano-mortars prepared with (**a**) superplasticizer and (**b**) surfactant as dispersive agent, as a function of F-T exposure (plain specimens have been added for reference).

Damage sensing capability during fracture as a function of F-T exposure is being illustrated based on the AE activity of un-modified, CNT-modified specimens prepared with superplasticizer or surfactant as dispersive agent (Figure 7a,b, respectively). The AE activity varied with the CNT content and dispersive agent in a non-monotonic way. Since pull-out of individual CNTs was expected to result in an enhanced acoustic response, a higher activity was indicative of a higher amount of well-dispersed tubes. Furthermore, it was observed that F-T cycling led to a decrease of the AE activity of all compositions. The observed reduction could be linked with the attenuation of the AE signal due to the water absorbed in the cementitious matrix during the thawing phase, as well as the existence of micro-cracks. Surfactant-containing mortars displayed a greater reduction of their AE activity after the exposure, which can be further linked with their more ductile failure pattern, assuming that brittle fracture creates more noise during crack propagation.

**Figure 8:**
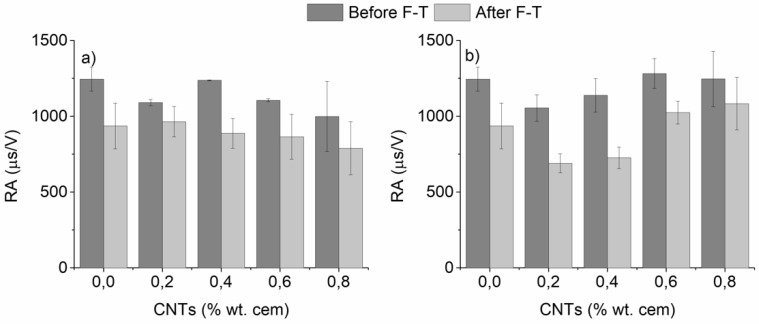
Rise time/amplitude (RA) values of nano-mortars prepared with (**a**) superplasticizer and (**b**) surfactant as a dispersive agent, as a function of F-T exposure (plain specimens have been added for reference).

**Figure 9:**
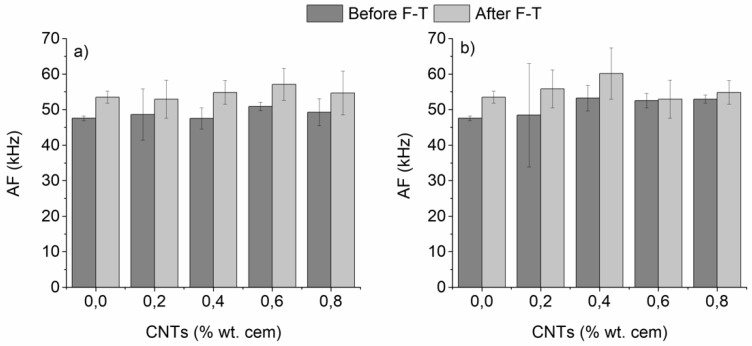
Average frequency of nano-mortars prepared with (**a**) superplasticizer and (**b**) surfactant as a dispersive agent, as a function of F-T exposure (plain specimens have been added for reference).

Two very interesting AE parameters used to evaluate the fracture mode of cementitious materials are the rise time/amplitude (RA) of the AE signal and the average frequency, counts/duration (AF) of the AE signal [49,50,51]. As stated before, in three-point bending tests mode I (tensile) cracks resulted in waveforms with low RA (in the vicinity of 2000 μs/V) and high AF (approximately 60 kHz), whereas mode II (shear) cracks corresponded to waveforms with higher RA (in the vicinity of 4000 μs/V) and lower AF (approximately 30 kHz) [49,50,51]. RA and AF values of the nano-modified mortars before and after F-T are plotted in Figure 8 and Figure 9, respectively.

As observed in Figure 8, F-T cycling resulted in a considerable reduction of RA on both un-modified and CNT-modified mortars prepared with superplasticizer/surfactant. Un-modified specimens exhibited a reduction of RA from approximately 1250 to 930 μs/V (26% drop). For CNT-modified mortars, the highest reduction was found after 0.4 wt.% nano-modification (28% and 37%, drop for superplasticizer and surfactant specimens, respectively). At the same time, as illustrated in Figure 9, all specimens presented an increase in the AF after F-T cycles from approximately 45 kHz to 55–60 kHz. The increase in un-modified specimens was approximately 12%, while nano-modified mortars presented a maximum increase of approximately 20%, which was found at intermediate CNT contents (0.4–0.6 wt.%), independent to the dispersive medium. 

Based on the obtained RA and respective AF values, the mode of fracture can be identified as tensile. The fact that the RA values drop, while the corresponding AF values increase, indicate a more tensile mode of fracture after F-T. This was observed, as aforementioned, in both un-modified and CNT-modified specimens, but became more intense in the case of CNT-modified mortars with 0.4–0.6 wt.% CNTs. As discussed throughout the current manuscript, F-T cycling resulted in the development of micro-cracks due to the expansion of absorbed water within the cementitious matrix and at the boundaries between the matrix and the aggregates. Modification of the cementitious matrix with CNTs was expected to lead to crack-bridging as well as pull-out at the nano-scale during bending, and in turn delayed fracture, providing the tubes were well dispersed and do not form agglomerates.

The fact that RA took its minimum value at 0.4–0.6 wt.% CNT content, where AF found its maximum after F-T implied that, at this range, CNTs were better dispersed and support pull-out and bridging mechanisms in a more controlled manner than in the case where the CNT content was low (0.2%), or very high (0.8%—extensive agglomeration). This is in line with previous data that found maximum improvement in flexural strength and respective drop of RA value of nano-modified mortars reinforced with 0.4 wt.% CNTs [18]. 

## 4. Conclusions

The current study investigated the durability of CNT-modified cementitious mortars after F-T cycling. CNTs were dispersed using two different types of agents, one belonging to the superplasticizer and the other to the surfactant family. Structural properties were assessed based on the dynamic elastic modulus, compressive strength and fracture energy of notched specimens under 3-point bending configuration. The obtained results led to the following conclusions:

- F-T exposure slightly enhanced the dynamic elastic modulus of un-modified and CNT-modified mortars prepared with a surfactant as a dispersive agent, while those prepared with superplasticizer present minor variations before and after F-T. This has been associated with enhanced hydration and compaction in specimens prepared with superplasticizer as a dispersant.
- F-T cycling did not significantly impact the compressive strength of CNT-modified specimens which was mainly determined by the microstructure of the specimens. The use of a superplasticizer as a dispersant facilitated the compaction of the specimens and enhanced their compressive strength prior and post F-T cycling.
- Nano-modification was beneficial for the durability of cementitious mortars exposed to F-T conditions since it resulted in an up to 73% higher fracture energy. The level of improvement was, however, dictated not only by the CNT content and dispersion efficiency but also by the nature of the dispersive agents. The dispersive agents controlled the size of micro-pores, the level of pressure built and, in turn, the way microcracks developed during the F-T cycling.
- Damage sensing activity was lower; however, it was still possible to identify a more tensile mode of fracture after F-T using critical AE indicators (RA and AF). This was more obvious in the case of nano-modified mortars with 0.4–0.6 wt.% CNTs. This fact was linked with better-dispersed nanotubes that supported pull-out/crack bridging mechanisms.
